# Follow-up management service and health outcomes of hypertensive patients in China: A cross-sectional analysis from the national health service survey in Jiangsu province

**DOI:** 10.3389/fpubh.2022.956711

**Published:** 2022-07-26

**Authors:** Mingyao Peng, Xinyi Shi, Lin Zhu, Zhonghua Wang

**Affiliations:** ^1^School of Health Policy Management, Nanjing Medical University, Nanjing, China; ^2^The Public Health Policy and Management Innovation Research Team, Nanjing Medical University, Nanjing, China; ^3^Center for Global Health, Nanjing Medical University, Nanjing, China

**Keywords:** follow-up management service, hypertension, health outcomes, control of blood pressure, self-reported health status

## Abstract

**Background:**

Hypertension is a major cause of early mortality worldwide. Health follow-up management services can encourage patients with hypertension to improve their health behavior and outcomes. However, a lack of studies on the relationship between specific factors of follow-up management and both subjective and objective health outcome among hypertensive patients exists. The current study investigated the relationship between service content, frequency, mode, and institutions of follow-up management and health outcomes among Chinese hypertensives.

**Methods:**

Data were obtained from the sixth National Health Service Survey (NHSS) of Jiangsu Province, which was conducted in 2018. Descriptive statistics were used to analyze the sample characteristics and the utilization of follow-up management services. Both multiple linear regression and logistic regression were used to estimate the association of follow-up management service and other factors with hypertensives' subjective and objective health outcomes.

**Result:**

Some respondents (19.30%) reported hypertension, and 75.36% of these patients obtained follow-up management services. Hypertensive patients' subjective health outcome self-reported health status and objective health outcome blood pressure (BP) control were found to be significantly associated with follow-up management services. The outcomes were both significantly improved by a high frequency of management services, a high level of follow-up providers, the mode of visiting healthcare facilities and/or calling, and receiving instructions on medication use. However, inquiring about patients' symptoms was negatively associated with self-reported health status and BP control. In addition, BP measurement was significantly and positively associated with hypertensive patients' self-reported health status; the patients receiving lifestyle guidance were more likely to have their BP levels under control.

**Conclusions:**

Hypertension management strategies should further focus on the frequency of healthcare follow-up management *via* categorization of the follow-up services and appropriate adjustment of service delivery modes to optimize health follow-up management for hypertensives further improve their outcomes. Meanwhile, complementary policies are also needed to address other socioeconomic factors that can promote good health conditions for hypertension patients.

## Introduction

Hypertension, one of the most frequent chronic disorders found in almost every country, has become a worldwide public health issue. According to data from the World Health Organization's Global Health Observatory [(GHO; (1))], hypertension affects around 1.13 billion people globally, resulting in millions of fatalities each year (2). In developed countries, hypertension affects about one-third of the individuals in the United States [(US); (3)], 26.3 million (37.5%) people in Japan (4), 30.5% of Koreans (5), and 30.1% of the French (6). In some developing countries, the prevalence of hypertension in Bangladesh is 25.7% (7), Malaysia 35.3% (8), and India 29.8% (9). According to the Nutrition and Chronic Diseases Status of Chinese Residents Surveys (2015–2019), hypertension affects 27.5% of adults (10). The tendency has continued to climb, constituting a major public health challenge for Chinese society (11–14). However, weaknesses in the effectiveness of hypertension control and management still exist. In the US, only 43.5% of hypertensives have their blood pressure (BP) under control (15), and these figures are 46.2% (5) and 15.0% (16) in Korea and Japan, respectively. In developing countries, the proportion of hypertensives with their BP under control in Bangladesh was 18.6% (7), in Malaysia 37.4% (8), and in India 17.7% (9). Furthermore, China has a very low rate of BP control as the percentage was only 13.8% (17).The hypertension guidelines of the American College of Cardiology/American Heart Association (ACC/AHA) from 2017 redefined the hypertension, systolic blood pressure (SBP) at 130 mmHg and diastolic blood pressure (DBP) at 80 mmHg as the criteria for diagnosis and target BP of treatment in adults (18). However, guidelines from high-income countries may not suit the global purpose (19). Due to China's suboptimal status, the hypertension guidelines do not currently adhere to these criteria (20). The International Society of Hypertension (ISH) released global practice guidelines for the treatment of hypertension in adults in 2020 (21) using a BP ≥ 140/90 mmHg as the minimum standard of care for hypertension. The same diagnostic criteria are used in the Chinese hypertension guidelines (2018) (11). Hypertension is a diagnosable condition that places the heart, brain, kidneys, and other organs in danger of damage. Heart failure atrial fibrillation and stroke are associated with higher BP levels (22–24). Furthermore, hypertension causes a significant financial and human resource strain on healthcare resources (2, 25). The health-related expenses of hypertension are increasing every year, confirmed by data from the World Bank. It is predictable that the social burden imposed by hypertension-related costs will grow in the future (26).

Although hypertension is difficult to cure, most hypertension pressure can be controlled. Once hypertension has occurred, it must be managed for the rest of an individual's life. Follow-up management can successfully regulate hypertensives' BP. Complications, such as severe cardiovascular and cerebrovascular illnesses; can be prevented with proper health management. In other nations, health management was established early and has a distinctive management structure. Studies have demonstrated that health management can lower the cost of healthcare, lessen the burden of disease, and enhance overall health (27). The development of health management in China lags behind that of other countries. The current models of health management in China mainly include the 4 health care homes and 8 health management model (4CH8), systematic model, self-help model, family doctor contracted service system model, etc. The effectiveness of these health management models still needs to be evaluated. Primary care physicians are at the vanguard of hypertension management, and these healthcare professionals should be in charge of hypertension identification, documentation, treatment, and long-term management services. The long-term management service is represented as a health follow-up management service, which is closely integrated with family doctor contracting service. In China, the family doctor contract service is the crucial part of the health management and basic public health service. The Chinese guidelines for hypertension prevention and control published in 2021 proposed standardized hypertension management in the community (10), and hypertension management teams should be established in community health service centers to provide long-term health follow-up management services for hypertensive patients (11). The follow-up management services included instructions about use of medications, monitoring of patients' BP levels, adverse responses to medications, and other factors, such as heart rate, lipids, blood glucose, target organ damage, and clinical illness to help doctors achieve the goal of evaluating patients' responses to their therapy, understanding patients' medication tolerance, examining whether BP treatment stabilizes risk factors, and establishing a good doctor–patient relationship based on mutual trust (11). Follow-up services also provide lifestyle coaching to patients with hypertension to reduce the risk factors with which they are faced. Furthermore, follow-up management is required at appropriate intervals to ensure that physicians are regularly informed of changes in the condition of hypertensive patients and guide patients' recovery.

Many studies have been conducted to investigate the association between follow-up management services and the health behaviors and status of chronic disease patients. Health follow-up management and healthy lifestyle treatments can increase diabetes knowledge for middle-aged and older schizophrenia patients with diabetes (28). Studies have demonstrated that comprehensive follow-up management can lead to a successful increase in patient compliance, minimization of hospitalization costs, and reduction in the occurrence of cirrhosis and hepatocellular cancer in patients with chronic liver disease (29). Diabetic patients benefit from community-based health follow-up management to better regulate their blood glucose levels. The effects of the intervention are long-term (30, 31). Health follow-up management can also help hemodialysis patients produces increases in their blood phosphorus levels, improvements in their physical and mental health, and improvements in their quality of life (32). With regard to hypertensive patients, some studies that health follow-up management service has led to a reduction in bad habits and an increase in good health behaviors in addition to a significant improvement in BP control (33). Group health care management is beneficial for increasing hypertensive patients' understanding of hypertension, whereas individual health care management is more useful for prompting behavior changes (34). The duration of follow-up visits and the intensity of health care providers' health follow-up are both positively connected with patients' enthusiasm for involvement (35). To boost health follow-up management more efficiently, several researchers have analyzed databases and mobile platforms for health follow-up management and evaluated this process's effect on facilitating remote monitoring and coaching for patients (2, 36).

Although existing studies have shown the effectiveness of health follow-up management in terms of leading to an improvement in disease control among hypertensive patients, a lack of studies on the relationship between specific factors of follow-up management of hypertension, such as service content, frequency, mode, or service institutions, and health outcomes exists. Furthermore, to our knowledge, the existing studies mainly focus on subjective or objective health outcomes, and few studies concerning a comparative analysis of the relationship between health follow-up management and both subjective and objective health outcomes among hypertensive patients are available. Compared to other literature, our research more comprehensively analyzes the effect of follow-up management on the health outcomes of patients with hypertension from multiple perspectives.

In this study, the association of follow-up management and both subjective and objective health outcomes was investigated using data from the National Health Service Survey (NHSS) in Jiangsu province in China. Specifically, the relationship between the service frequency, content, mode and institution of health follow-up management, and other relevant factors and health outcomes of hypertensive patients were evaluated. Our findings have important implications for policies aimed at improving hypertension prevention and treatment in China and providing evidence for optimizing the health follow-up management services of chronic diseases.

## Materials and methods

### Data source

Data were obtained from the sixth NHSS (2018) of Jiangsu Province. The NHSS is a 5-yearly survey conducted by the Center for Health Statistics and Information of the National Health Commission since 1993. In the survey, samples were obtained from 156 counties (cities and districts) in 31 provinces through multi-stage stratified random sampling. In each county, five towns (streets) were randomly selected, two villages (neighborhood committees) were randomly selected in each town, and then 60 households were randomly selected in each village. A total population of nearly 300,000 from 93,600 households nationwide was examined. Jiangsu province is a densely populated area in Eastern China and is one of the highest comprehensive development levels in the country with a population of 84.748 million. A total of 11,550 individuals from 3,600 household samples were randomly selected from 19 counties to participate in the survey in 2018. The supervisor directed and checked each step of the survey for quality control. Surveyors were trained to conduct qualified face-to-face interviews. The district survey manager checked the questionnaire at the end of each day to avoid missing information or logical errors (37). In addition, 5% of the sampled households were randomly selected for revisits to check data accuracy. Therefore, the data retrieved are all effective. The total sample in the study contained 2,229 respondents who suffered from hypertension. Thus, the prevalence of hypertension in Jiangsu Province was 19.30% in 2018.

The data in the NHSS covered detailed personal information, including demographic and socioeconomic characteristics (gender, year of birth, education status, employment status, marital status, area of residence, depression, self-reported health status, and chronic diseases, and others) of those hypertensive patients who received or did not receive follow-up management services. The survey also included questions on hypertensive patients' utilization of health follow-up management services and health behaviors (smoking, drinking, exercise, medication, and BP measurement frequency).

### Variable selection

#### Dependent variables

The dependent variables included two kinds of health outcomes: (1) self-reported health status and (2) control of BP. Self-reported health status is a subjective outcome, and control of BP is an objective one. Self-reported health status is a well-known measure for assessing the quality of life (QoL), and it is widely being applied in the evaluation for chronic illnesses patients (38). The Euro-Qol Visual Analog Scale (EQ-VAS) is a vertical visual scale 20 cm long. The top A score of 100 at the top represents the “best perceived health” and a score of 0 at the bottom represents the “worst perceived health.” The EQ-VAS score is directly available from the survey, and the scores are sensitive and easily reflect small changes in survival quality. It is a widely used measure of self-reported health status that allows for the evaluation of overall health scores and contributes to the subjective perception of patients with chronic diseases about their health condition (39). Control of BP is indicated by whether the last measured BP value was normal or not.

#### Independent variables

The data from the NHSS includes detailed information about the respondents, and the questionnaire included questions about the demographic and socioeconomic characteristics of those hypertensive patients, including their gender, marital, educational, and employment statuses, and others. Furthermore, the questionnaire also investigated hypertensive patients' utilization of health follow-up management services: follow-up frequency (1. 1 time; 2. 2 times; 3. 3 times; 4. 4 times; 5. at least 5 times), follow-up institutions (1. village clinic/private clinic/community healthcare service station; 2. township hospital/community healthcare service center; 3. medical and healthcare organization above the county level; 4. health management institution; 5. others), follow-up modes (1. households and home visiting; 2. going to medical institutions; 3. calling; 4. others), and follow-up contents (1. BP measurement; 2. lifestyle guidance; 3. inquiring about symptoms; 4. Instruction on medications). The service frequency and the contents always represented the service items or standardization of follow-up management. Furthermore, the mode and institution of follow-up management services meant difference in the organization and model of health management. Detailed descriptions of the variables included in the study can be found in Table 1.

**Table 1:** Description of variables.

**Variable**	**Description**	**Survey questions**
**Dependent variable**		
Self-reported health status (VAS score)		Question: How would you rate your health today on a scale of 0 (worst possible health) to 100 (best possible health)?
Control of blood pressure	0, No	Question: Is your current (last measured) blood pressure normal.
	1, Yes	
**Independent variable**		
Follow-up frequency	0, No follow-up	Question: How many times did medical staff follow up your hypertension in the past 12 months?
	1, 1 time	
	2, 2 times	
	3, 3 times	
	4, 4 times	
	5, At least 5 times	
Follow-up institution	1, Village clinic/Private clinic/Community health service station	Question: From which institution do you obtain hypertension follow-up service most?
	2, Township hospital/Community health services center	
	3, Medical and health organization above the county level	
	4, Health management institution	
	5, Other	
Follow-up mode	1, Households and home visiting	Question: What kind of hypertension follow-up service did you get recently?
	2, By going to medical institution	
	3, By calling	
	4, Other	
Follow-up content		
Blood pressure measurement	0, No	Question: What were the main contents of your last hypertension follow-up? (Options include:blood pressure measurement, lifestyle guidance, symptom inquiry, medication instructions).
	1, Yes	
Lifestyle guidance	0, No	
	1, Yes	
Inquiring symptoms	0, No	
	1, Yes	
Instruction on medication	0, No	
	1, Yes	
Demographic variable		
Gender	1, Male	
	2, Female	
Age		Question: Year of birth.
Education status	1, Less than lower secondary	Question: What is your education level?
	2, Upper secondary /vocational training	
	3, Tertiary	
Employment status	0, Unemployed	Question: What's your employment status?
	1, Employed	
	2, Retired	
Marital status	0, Not married (single, widowed, divorced, or other)	Question: What's your marital status?
	1, Married	
Area of residence	0, Rural	Question: What's your registered type?
	1, Urban	
Depression	1, Not depressed	Question: What is the extent of your anxiety or depression?
	2, Moderately depressed	
	3, Severely depressed	
BMI		BMI is the weight divided by the square of the height.
**Health Behaviors**		
Smoking	0, No	Question: Are you currently a smoker?
	1, Yes	
Drinking	0, No	Question: Do you currently drink alcohol?
	1, Yes	
Exercise	1, Less than once a week	Question: In the past 30 days, how many times do you exercise consciously every week?
	2, At least once a week	
Medication	0, Erratically 1, Regularly	Question: How are you taking antihypertensive drugs? (The answer is “take regularly,” “take occasionally or when necessary,” “take sometimes”, and “never take,” and the last three were considered erratically).
Blood pressure measurement frequency	1, Weekly 2, Monthly 3, Once every 1 to 3 months 4, Once every 3 to 6 months 5, Once every6 to 12 months 6, More than 12 months	Question: When was the last time you had your blood pressure measure? (The answer is “within 1 week,” “within 1 month,” “more than 1 month,” “more than 3 months,” “more than 6 months,” and “more than 12 months,” were considered “weekly,” “monthly,” “Once every 1 to 3 months,” “Once every 3 to 6 months,” “Once every6 to 12 months,” and “More than 12 months”).

*VAS, visual analog scale*.

### Statistical analysis

Initially, the characteristics of hypertensive patients received vs. those who have not received health follow-up management service are described. The prevalence of categorical variables and the mean and standard deviation of continuous variables were calculated after which descriptive statistics were used to show the prevalence of each follow-up management service variable with 95% confidence intervals (CI). Finally, multiple linear regression models and binary multivariate logistic regression were applied to evaluate the association between health follow-up management service and other factors with self-reported health status and BP control of hypertensive patients, respectively. Every regression was evaluated by two models, among which follow-up frequency, service institutions, modes, and service contents were included in the first model, and demographic variables of hypertensive patients were added to the second model. Statistical analyses were performed using STATA software (v.16.0; STATA Corp, College Station, TX, USA).

## Results

### Characteristics of hypertensives

The total sample consisted of 2229 respondents who reported that they had been diagnosed with hypertension. Among all hypertensive patients, 75.37% (1,680) received health follow-up management service, and 24.63% (549) did not receive the service. Table 2 shows the descriptive statistics of characteristics of the hypertensive patients who had vs. those who had not received follow-up management services. The average score of the self-reported health status of follow-up patients was 74.57, while that of non-follow-up patients was 73.87. The rate of BP control of followed-up patients with hypertension (75.36%) was higher than that of patients who had not been followed up (67.58%). The follow-up rate was slightly higher for women (77.33%) than men (73.41%) and rural residents (78.90%) vs. urban residents (73.10%). Follow-up hypertensive patients' average age (64.41 years) was slightly higher than the non-follow-up group (63.34 years).

**Table 2:** Sample characteristics of hypertensives.

	**Total**	**Follow-Up**	**Not follow-up**	***p-value***
	**(*****n*** = **2229)**	**(*****n*** = **1680)**	**(*****n*** = **549)**	
Dependent variable				
Self-reported health status (mean ± Sd)	74.40 ± 0.32	74.57 ± 0.36	73.87 ± 0.68	0.352
Control of blood pressure (%)				
Yes	73.44	75.36	67.58	0.000***
No	26.56	24.64	32.42	
Demographic variable				
Age (mean ± Sd)	64.15 ± 10.99	64.41 ± 10.47	63.34 ± 12.43	0.049**
Gender (%)				0.031**
Male	50.11	48.81	54.1	
Female	49.89	51.19	45.9	
Area of residence (%)				0.002***
Rural	39.12	40.95	33.52	
Urban	60.88	59.05	66.48	
Employment status (%)				<0.001***
Unemployed	27.5	29.76	20.58	
Employed	36.38	36.37	36.43	
Retired	36.12	33.87	42.99	
Education status (%)				<0.001***
Less than lower secondary	79.95	82.8	71.22	
Upper secondary /vocational training	15.7	13.39	22.77	
Tertiary	4.35	3.81	6.01	
Marital status (%)				0.496
Not married	14.18	14.46	13.3	
Married	85.82	85.54	86.7	
Depression (%)				0.615
Not depressed	89.82	89.88	89.62	
Moderately depressed	9.55	9.41	10.02	
Severely depressed	0.63	0.71	0.36	
BMI (mean ± Sd)	24.43 ± 3.50	24.44 ± 3.45	24.39 ± 3.66	0.788
Behavior variable				
Smoking (%)				0.048**
Yes	24.18	23.15	27.32	
No	75.82	76.85	72.68	
Drinking (%)				0.183
Yes	31.12	31.37	34.43	
No	67.88	68.63	65.57	
Exercise (%)				0.032**
Less than once a week	42.89	41.61	46.81	
At least once a week	57.11	58.39	53.19	
Medication (%)				<0.001***
Yes	87.57	89.82	80.69	
No	12.43	10.18	19.31	
Blood pressure measurement frequency (%)				<0.001***
Weekly	44.01	46.25	37.16	
Monthly	32.03	33.87	26.41	
Once every 1 to 3 months	12.16	11.61	13.84	
Once every 3 to 6 months	7.09	6.25	9.66	
Once every 6 to 12 months	2.24	1.25	5.28	
More than 12 months	2.47	0.77	7.65	

**Implies p-value < 0.05. ***Implies p-value < 0.001, Sd, standard deviation.

Hypertensive patients who received health follow-up management services had significantly fewer negative health behaviors, such as smoking (23.15% and 27.32% for received and did not receive, respectively) and alcohol consumption (31.37% and 34.43%, respectively) than those who did not receive the service. Over half (58.39%) of the hypertensive patients who received health follow-up management services participated in daily exercise at least once a week, which was significantly higher than those who did not receive the service (53.19%). Those who received health follow-up management services demonstrated a higher prevalence of medication adherence (89.82%) when compared with patients who did not receive the service (80.69%). Furthermore, hypertensive patients who received health follow-up management services also measured their BP more regularly than those not receiving the service.

### Health follow-up management service in hypertensives

Table 3 presents the prevalence of different health management service among 1,680 hypertensive patients who received health follow-up management services. As shown in Table 3, most hypertensives were followed up every months, 57.86% of patients receive health follow-up management service at least 5 times a year, and 19.58% received health follow-up management service 4 times a year. Health follow-up management services were mainly provided by the village clinic/private clinic/community health service station (49.29%) and township hospital/community health services center (43.45%). The proportion of hypertensive patients who received health follow-up management services by going to the medical institution (61.61%) was higher than that of those receiving services in their household with home visits (27.5%), calling (7.44%), or by other means (3.45%). The follow-up management provided hypertensive patients with BP measurements (97.26%), lifestyle guidance (86.07%), symptom inquiries (86.85%), and medication instruction (91.61%).

**Table 3:** Prevalence of different health management services among hypertensives who received follow-up.

**Variable/categories**	**Prevalence (%)**	**95% CI**		**n**
Follow-up frequency				
1 time	6.31	0.0519	0.0758	106
2 times	7.56	0.0634	0.0634	127
3 times	8.69	0.0739	0.1014	146
4 times	19.58	0.1771	0.2156	329
At least 5 times	57.86	0.5545	0.6023	972
Follow-up institution				
Village clinic/Private clinic/Community health service station	49.29	0.4687	0.5171	828
Township hospital/Community health services center	43.45	0.4107	0.4586	730
Medical and health organizations above the county level	4.52	0.0358	0.0563	76
Health management institution	0.06	0.0563	0.0033	1
Other	2.68	0.0196	0.0357	45
Follow-up mode				
Households and home visiting	27.5	0.2538	0.2970	462
By going to a medical institution	61.61	0.5923	0.6394	1035
By calling	7.44	0.0623	0.0880	125
Other	3.45	0.0263	0.0444	58
Follow-up Content				
Blood pressure measurement				
Yes	97.26	0.9636	0.9799	1634
No	2.74	0.0201	0.0364	46
Lifestyle guidance				
Yes	86.07	0.8432	0.8769	1446
No	13.93	0.1231	0.1568	234
Inquiring symptoms				
Yes	86.85	0.8513	0.8843	1459
No	13.15	0.1157	0.1487	221
Instruction on medication				
Yes	91.61	0.9018	0.9289	1539
No	8.39	0.0711	0.0982	141

### Multivariate regression results

#### Linear regression of self-reported health status

In this paper, we use the variance inflation factor to test the covariance between variables, and it is generally considered that VIF>10 indicates the existence of multicollinearity between variables. The test results show that the maximum value of VIF in the two models is 4.53, which indicates that there is no multicollinearity problem between variables. Table 4 shows the linear regressions result of the self-reported health status among the patients with hypertension who received health follow-up management services. Both Models 1 and 2 indicate that VAS scores were significantly associated with the follow-up management factors. When receiving the service once a year was taken as the control group, the increased frequency of follow-up did have a significant role in improving VAS score, 2 times (coefficient [coef.] = 18.250, 5.868), 3 times (coef. = 15.659, 4.541), 4 times (coef. = 15.803, 4.193), and at least 5 times (coef. = 18.036, 6.113). Obtaining the service from village clinic/private clinic/community health service station as a reference, the health status scores of the hypertensive patients receiving health follow-up management services from township hospital/community health services center (coef. = 5.420), medical and health organization above the county level (coef. = 6.800), and other institutions (coef. = 10.814) were significantly higher in Model 1, while in the second model, only receiving the service from the township hospital/community health services centers showed significantly higher scores of self-reported health status (coef. = 1.394). The follow-up mode of going to the medical institution (coef. = 7.038, 2.240) and calling (coef. = 13.048, 2.807) demonstrated significantly higher VAS scores than the mode of households and home visiting. In both two models, providing patients with follow-up services for BP measurements (coef. = 39.822, 13.602) and medication instruction (coef. = 8.875, 3.616) led to a significant improvement in self-reported health scores; however, the service involving lifestyle guidance had no significant effect. In model 2, the service that inquired about symptoms (coef. = −2.510) was inversely correlated with the health status scores.

**Table 4:** Linear regression of self-reported health status among hypertensives received follow-up (*n* = 1680).

	**Model 1**				**Model 2**		
	***Coef***.	***Std.Err***.	***95%CI***		***Coef***.	***Std.Err***.	***95%CI***
Follow–up frequency, ref.: 1 time						
2 times	18.250***	2.077	(14.177,22.324)		5.868***	1.844	(2.252,9.485)
3 times	15.659***	2.009	(11.719,19.599)		4.541**	1.781	(1.048,8.035)
4 times	15.803***	1.757	(12.356,19.250)		4.193***	1.595	(1.064,7.322)
At least 5 times	18.036***	1.595	(14.909,21.164)		6.113***	1.457	(3.255,8.971)
Follow–up institution, ref.: Village clinic/Private clinic/Community health service station						
Township hospital/Community health services center	5.420***	0.876	(3.702,7.138)		1.394*	0.803	(−0.181,2.968)
Medical and health organizations above the county level	6.800***	2.046	(2.786,10.813)		1.764	1.755	(−1.678,5.206)
Health administration institution	6.683	17.140	(−26.935,40.301)		−1.857	14.454	(−30.208,26.493)
Other	10.814***	3.366	(4.213,17.416)		4.172	2.851	(−1.421,9.764)
Follow–up mode, ref.: Households and home visiting						
By going to a medical institution	7.038***	0.957	(5.161,8.915)		2.240***	0.831	(0.609,3.870)
By calling	13.048***	1.689	(9.735,16.360)		2.807*	1.528	(−0.190,5.803)
Other	4.194	3.033	(−1.755,10.143)		0.976	2.561	(−4.046,5.998)
Follow–up content						
Blood pressure measurement, ref.: No	39.822***	1.878	(36.139,43.505)		13.602***	2.092	(9.498,17.705)
Lifestyle guidance, ref.: No	2.342	1.582	(−0.762,5.446)		2.078	1.338	(−0.545,4.701)
Inquiring symptoms, ref.: No	0.048	1.757	(−3.398,3.494)		−2.510*	1.482	(−5.416,0.396)
Instruction on medication, ref.: No	8.875***	1.870	(5.207,12.543)		3.616**	1.610	(0.459,6.774)
Age					0.222***	0.031	(0.162,0.282)
Gender, ref.: Male					1.278*	0.731	(−0.156,2.713)
Area of residence, ref.: Rural					2.813***	0.873	(1.101,4.525)
Employment status, ref.: Unemployed						
Employed					10.349***	0.979	(8.429,12.268)
Retired					5.166***	0.987	(3.231,7.101)
Education status, ref.: Less than lower secondary						
Upper secondary /vocational training					2.234**	1.062	(0.152,4.317)
Tertiary					1.856	1.876	(−1.824,5.536)
Marital status, ref.: Not married					3.793***	1.022	(1.789,5.797)
Depression, ref.: Not depressed						
Moderately depressed					−12.883***	1.218	(−15.272,−10.495)
Severely depressed					−16.989***	4.150	(−25.129,−8.850)
BMI					1.068***	0.086	(0.900,1.237)

*Implies p–value < 0.1. **Implies p–value < 0.05. ***Implies p–value < 0.001; ref, reference category; CI, confidence interval.

Among the demographic factors in model 2, the VAS scores correlated significantly with age, gender, employment, education, area of residence, marriage, depression, and body mass index (BMI). The self-reported health status was positively correlated with age (coef. = 0.222) and BMI (coef. = 1.068). Female (coef. = 1.278) and married (coef. = 3.793) hypertensive patients had relatively better self-reported health statuses than men and not married patients. Hypertensive patients living in urban (coef. = 2.813) had higher VAS scores than those in rural. When compared with the unemployed patients, the employed (coef. = 10.349) and retired patients (coef. = 5.166) had significantly better self-reported health status. The VAS scores of hypertensive patients with intermediate (upper secondary/ vocational training) education levels (coef. = 1.278) were significantly higher than those with low education levels. The result also shows that the VAS scores were significantly lower among moderately depressed (coef. = −12.883) and severely depressed patients (coef. = −16.989).

#### Logistic regression for control of BP

The binary multivariate logistic regression results for BP control are shown in Table 5. Since only one patient received follow-up service provided by the healthcare management institution, this sample was deleted from the logistic regression model for estimation accuracy.

**Table 5:** Logistic regression model on control of blood pressure in hypertensives received follow–up (*n* = 1679).

	**Model 1**				**Model 2**		
	***OR***	***Std.Err***.	***95%CI***		***OR***	***Std.Err***.	***95%CI***
Follow–up frequency, ref.:1 time						
2 times	0.868	0.239	(0.506,1.489)		0.801	0.239	(0.447,1.436)
3 times	0.991	0.267	(0.585,1.680)		0.955	0.277	(0.541,1.686)
4 times	1.328	0.319	(0.829,2.127)		1.309	0.347	(0.778,2.201)
At least 5 times	1.833***	0.404	(1.190,2.823)		1.626**	0.398	(1.007,2.625)
Follow–up institution, ref.: Village clinic/Private clinic/Community health service station						
Township hospital/Community health services center	2.039***	0.258	(1.591,2.614)		1.509***	0.209	(1.150,1.980)
Medical and health organizations above the county level	1.050	0.285	(0.617,1.786)		0.767	0.215	(0.443,1.329)
Health administration institution	1.000	(empty)			1.000	(empty)	
Other	4.245***	2.244	(1.506,11.964)		3.737**	1.973	(1.328,10.516)
Follow–up mode, ref.: Households and home visiting						
By going to a medical institution	1.627***	0.215	(1.255,2.107)		1.437***	0.197	(1.098,1.881)
By calling	1.485*	0.355	(0.929,2.374)		0.973	0.255	(0.582,1.627)
Other	0.757	0.312	(0.337,1.698)		0.665	0.273	(0.297,1.489)
Follow–up content						
Blood pressure measurement, ref.: No	0.685	0.180	(0.409,1.146)		0.450**	0.179	(0.206,0.983)
Lifestyle guidance, ref.: No	1.458*	0.321	(0.947,2.246)		1.554*	0.350	(1.000,2.416)
Inquiring symptoms, ref.: No	0.636*	0.162	(0.386,1.048)		0.604*	0.157	(0.362,1.007)
Instruction on medication, ref.: No	1.796**	0.461	(1.086,2.970)		1.668*	0.444	(0.990,2.812)
Age					1.007	0.005	(0.996,1.017)
Gender, ref.: Male					1.138	0.142	(0.891,1.453)
Area of residence, ref.: Rural					1.550***	0.226	(1.164,2.064)
Employment status, ref.: Unemployed						
Employed					0.921	0.147	(0.674,1.259)
Retired					1.202	0.211	(0.853,1.694)
Education status, ref.: Less than lower secondary						
Upper secondary /vocational training					1.532**	0.300	(1.044,2.249)
Tertiary					1.305	0.470	(0.644,2.645)
Marital status, ref.: Not married					1.276	0.216	(0.917,1.777)
Depression, ref.: Not depressed							
Moderately depressed					0.574***	0.106	(0.399,0.826)
Severely depressed					0.517	0.315	(0.157,1.706)
BMI					0.993	0.014	(0.966,1.022)

*Implies p–value < 0.1. **Implies p–value < 0.05. ***Implies p–value < 0.001; ref, reference, OR odd ratio; ref, reference category; CI, confidence interval.

In model 1, patients with hypertension had a significantly higher likelihood of BP control when the frequency of service was at least five times a year in contrast with the frequency of once a year (odds ratio [OR] = 1.833). The hypertensive patients who received follow-up services from township hospitals/community health service centers and other institutions showed a significant and positive impact on BP control. As shown in Table 5, the probability of keeping BP under control among patients who received service from township hospital/community health services center and other institutions were 2.039 and 4.245 times, respectively, that of patients obtaining follow-up management service from a village clinic/ private clinic. The likelihood of BP control was significantly enhanced by providing follow-up service of lifestyle guidance (OR = 1.458) and instructing patients on medication (OR = 1.796). However, the service of inquiring about symptoms produced a significantly negative impact on BP control (OR = 0.636). The follow-up mode of going to a medical institution (OR = 1.627) and calling (OR = 1.485) produced a significantly higher likelihood of controlling BP when compared with households and home visiting.

When the regression included demographic factors, the mode of telephone follow-up service showed an insignificant influence on the control of BP in model 2 and the likelihood of BP under control was significantly lower as a result of providing a follow-up service of blood pressure measurement (OR = 0.450). with regard to demographic factors. Patients who lived in urban areas were more likely to keep their BP in the normal range (OR=1.550). The likelihood of BP control among patients with intermediate (upper secondary/ vocational training) education level (coef. = 1.278) were significantly higher than those with low education level. In addition, depression and anxiety are adverse factors that affect BP control, especially, moderate anxiety and depression were found to be significantly detrimental to the likelihood of BP control (OR = 0.574). However, the negative impact of severe anxiety and depression on BP control was not significant.

## Discussion

With the ongoing development of the Chinese economy and the aging of population, the prevalence of hypertension has dramatically risen. Hypertension must be managed by therapy and to keep BP within normal ranges and to delay and control its progression as much as possible to avoid its negative effects. Despite the fact that health follow-up management services have become an important aspect of hypertension control, few researchers have investigated the correlation between the service content, frequency, mode, and institution of follow-up management and hypertensive patients' health status. This study, using NHSS data from Jiangsu province in 2018, assessed the association between the utilization of health follow-up management services and subjective and objective health outcomes of hypertensive patients.

In this study, 19.30% of the respondents reported hypertension, which was lower than the national prevalence of hypertension (10). This finding may have been caused by lack of awareness by some patients of their hypertension because they had not been diagnosed and/or did not self-report their condition. According to previous studies, less than half of those with hypertension are aware of their disease (13, 14). More than three-quarters of the patients reported that they had received health follow-up management services for hypertension during the past year. This finding confirms widespread implementation of health follow-up management service among hypertensive patients in China. “The Health China Action (2019–2030)” also requires primary healthcare institutions to provide basic and standardized medical health management services for hypertensive patients (40, 41). Consistent with the previous study (42), our study demonstrated that a higher proportion of patients in rural areas received health follow-up management services than those in urban residents. When compared with urban communities, the residential characteristics and neighborhood relationships in rural China make it easier for primary healthcare institutions, such as village health centers, to provide health follow-up management services, allowing for greater coverage of health follow-up management in rural area.

The hypertensives who received follow-up management services had better lifestyle habits and health-related behaviors as demonstrated by less smoking, less excessive drinking, more exercise, and more regular medication use and BP monitoring. As shown in Table 3, follow-up management offered most patients lifestyle guidance and knowledge about medications, which may have effectively promoted their health awareness and self-management behaviors. Previous research has demonstrated that health care providers' lifestyle recommendations can help patients improve their behavior (43, 44). Our results indicate that follow-up hypertensive patients' self-reported health scores were slightly higher than those of non-follow-up patients and that their BP control rate was also significantly higher than that of the non-follow-up group. The health status of the follow-up group was generally better than the non-follow-up group. Thus, implementing the public health service system of family doctor contracting and taking advantage of health follow-up managementin China are important for patients with hypertension in terms of improving their health outcomes (45, 46).

Multiple regression results for follow-up factors demonstrated that various follow-up variables were significantly associated with hypertensive patients' health outcomes. Specifically, the frequency of follow-up visits, the service institutions, the mode of follow-up, and the content of follow-up services were all associated with self-reported scores and BP control. Patients who had more follow-up services per year had higher self-reported scores than those who only had one visit per year, and those who had at least five visits per year were more likely to maintain normal BP. It suggests that the frequency of follow-up visits affects not only the subjective health status, but also objective physiological health indicators of hypertensive patients. Therefore, to ensure that hypertensive patients have favorable health outcomes, strategies of chronic disease management should pay more attention to the frequency of health follow-up management. According to our findings, not less than five times a year is appropriate for hypertensive patients. Patients who receive follow-up services from a higher-level health provider were found to be more likely to have higher self-reported health scores and control their BP better than those who received follow-up services from a clinic or health service station. This difference might have occurred because higher-level healthcare institutions have better healthcare service capacity than primary care facilities and may provide hypertensive patients with more effective health follow-up management services. Therefore, the government should consider the healthcare service capacity of different medical institutions, categorize follow-up management services according to the health status of hypertensive patients, and make the best use of health resources to improve the overall health outcomes.

Hypertensives receiving follow-up service showed better health outcomes in mode of going to the institution or calling than those receiving home visitations. A previous research study also showed that the effectiveness of health management is influenced by the structure and intensity of different delivery modes (44). However, the current health follow-up service is mainly conducted by primary health care providers in the form of home visits or patient visits to institutions. Therefore, it is necessary to further enrich the delivery modes of health follow-up management services; the mode of telephone or e-health may also be an effective way of delivering follow-up services that help doctors conveniently monitor hypertensive patients' BP and make it easier for patients to seek aid and counsel (47) so as to improve the health status of hypertensive patients. BP measurements were significantly and positively associated with a hypertensive patient's self-reported health status but did not correlate with the likelihood of BP control. However, those who receive lifestyle coaching are more likely to have controlled BP. Lifestyle guidance may enable hypertensive patients to be less likely to be exposed to risk factors such as a high-salt diet or a lack of exercise, which is a result similar to that in previous studies (43, 48) In this study, patients who accepted instructions on medication not only showed an improvement in their health status scores but also were more likely to have their BP under control. This finding highlights the significance of medication instruction in health follow-up management services. Targeted instruction concerning medication can standardize patients' medication appropriateness and adherence thus, effectively improving health outcomes for hypertensives. However, inquiring about patients' symptoms is counterproductive to BP control and self-reported health status; the possible reason for this finding is that inappropriate frequency or method of inquiry about symptoms may cause physical and psychological distress, which eventually influences self-reported health and even BP control. Therefore, the frequency and manner of inquiry should be considered during the health follow-up process so as to alleviate the negative impact on hypertensive patients' health status.

The findings also indicate that health outcomes were significantly associated with the hypertensive patients' demographic characteristics factors, such as gender, education level, marriage, anxiety and depression. Females always present superior health literacy over males (49, 50) and usually have better health conditions. Being married has a positive impact on their spouse's lifestyle, and the partner can also play a supervisory role, encouraging hypertensive patients to follow good habits and self-management behaviors (51–53). In contrast to previous studies (12), our study demonstrated no significant influence of employment status on BP control, but the hypertensive patients who worked and retired had higher self-reported health scores than those who were unemployed. This finding may be due to the fact that unemployment may bring hypertensive patients mental stress and confusion, and thus lead to a decrease in self-reported health status. Patients with intermediate levels of education scored higher on self-reported health status and were more likely to have control over their BP than those with low levels of education, but this was not the case for those with high levels of education. This implies that a positive non-linear correlation between education and health status among hypertensives patients exists, and secondary education level demonstrated the most significant effect. Patients with hypertension in urban areas were more likely to keep their BP in the normal range and have higher self-reported health scores than rural residents, which may be due to the great gap in the accessibility, quantity and quality of healthcare resources between rural and urban areas in China and medication related to urban-rural income disparities (54). Therefore, strategies to reduce the gap of socioeconomic development and optimize the distribution of health resources between rural and urban area should be implemented to improve accessibility of healthcare services in rural areas and to ameliorate health outcomes of rural hypertensive patients.

To sum up, intervention strategies at the providers, community, and individual levels are needed to improve the health outcomes of people with hypertension. At the provider level, the two-way referral mechanism has been continuously strengthened with the development of the Medical Alliance in China, and this mechanism should be practically applied to hypertension management. Through the optimization of service procedures and health resources allocation, the two-way referrals can increase the efficiency of follow-up management, which in turn improved the health outcomes. Meanwhile, general practitioners who offer primary health care have a vital role in follow-up management services of hypertension, but most of them have a poor education and training level in China (54, 55). As a result, the quality of health follow-up management services is still not very good. Therefore, policy efforts should further focus on increasing education and/or training levels of primary general practitioner to further improve their service ability. Additionally, the goal of health follow-up management is to keep track of chronic patients and promote them to increase their health awareness and outcomes. Patients' health literacy also has an impact on their compliance with follow-up management (56). Therefore, strengthening community health education should be an effective way to help patients develop a correct awareness of hypertension, and establish an active health habit in favor of compliance with follow-up management services.

Some limitations of our study must be acknowledged. First, the data analyzed in the study were cross-sectional, making it impossible to draw any causal inferences. Longitudinal investigations are needed in the future to validate the accuracy of the findings. The data were collected using an interview instrument with standardized questions and self-reported replies, prone to homozygous bias and self-reported bias (57). Last but not the least, NHSS data lack precise physiological indications, such as SBP/DBP values, and do not distinguish between primary hypertension and secondary hypertension; thus, in-depth analysis of detailed subgroup differences in the results was not possible.

This current study implies that health follow-up management services were significantly associated with hypertension patients' subjective and objective health outcomes. A high frequency of management services, high level of follow-up providers, mode of visiting health facilities and/or calling, and medication instructions were significantly positively associated with their self-reported health status and the likelihood of BP control. However, inquiring about patients' symptoms was negatively correlated with self-reported health status and BP control. BP measurements was significantly positively associated with hypertensive's self-reported health status and providing lifestyle guidance were more likely to have their BP under control. Thus, the hypertension management strategy should focus on the frequency of health follow-up management, categorizing the follow-up services and appropriate adjustment of services delivery modes to optimize health follow-up management for hypertensives to further improve patients' health outcomes. Furthermore, some demographic factors, such as gender, marriage, education level, residence area, and others, also produce a significant impact on hypertensives' health outcomes. Thus, complementary policies are required to address these socioeconomic determinants to promote adequate healthcare conditions for hypertension patients. Finally, further research, especially longitudinal studies, is needed to better understand the causal links between follow-up management and the factors that influence it.

## Data availability statement

The data analyzed in this study is subject to the following licenses/restrictions. The datasets used in the current study are not publicly available due to the confidential policy but are available from the corresponding author on reasonable request. Requests to access these datasets should be directed to wzh04@njmu.edu.cn.

## Ethics statement

Written informed consent was obtained from the individual(s) for the publication of any potentially identifiable images or data included in this article.

## Author contributions

MP led the analysis of the data and wrote the first draft of the manuscript. ZW contributed to the study design, interpretation of the data, and helped in the writing of the final draft of the manuscript. XS and LZ helped in data analysis and contributed in writing. All authors have read and agreed to the published version of the manuscript.

## Funding

This study was funded by the major research project of philosophy and social sciences in colleges and universities in Jiangsu Province (no.2021SJZDA148). The funding bodies were not involved in the design of the study, or data collection, analysis, and interpretation or in writing the manuscript.

## Conflict of interest

The authors declare that the research was conducted in the absence of any commercial or financial relationships that could be construed as a potential conflict of interest.

## Publisher's note

All claims expressed in this article are solely those of the authors and do not necessarily represent those of their affiliated organizations, or those of the publisher, the editors and the reviewers. Any product that may be evaluated in this article, or claim that may be made by its manufacturer, is not guaranteed or endorsed by the publisher.
